# Management and surveillance of metastatic giant cell tumour of bone

**DOI:** 10.3389/pore.2025.1611916

**Published:** 2025-02-19

**Authors:** David Fellows, Julia Kotowska, Thomas Stevenson, Jennifer Brown, Zsolt Orosz, Ather Siddiqi, Duncan Whitwell, Thomas Cosker, Christopher L. M. H. GIbbons

**Affiliations:** ^1^ Oxford Sarcoma Service, Nuffield Orthopaedic Centre, Oxford, England, United Kingdom; ^2^ Trauma and Orthopaedics, Hampshire Hospitals NHS Foundation Trust, Basingstoke, United Kingdom; ^3^ Trauma and Orthopaedics, Institute of Naval Medicine, Alverstoke, United Kingdom

**Keywords:** giant cell tumor of bone, pulmonary metastasis, sarcoma, orthopaedic oncology, surveillance

## Abstract

Giant cell tumour of bone (GCTB) is viewed as a benign, locally aggressive primary bone tumour with metastatic potential. Current management is surgery with bone curettage or resection and systemic therapy with denosumab. Diagnosis is confirmed histologically prior to surgery, with staging for pulmonary disease, as pulmonary metastases (PM) reportedly occur in <8%. This study aimed to assess incidence, surveillance and management of PM in patients with GCTB, with histopathological review. A retrospective audit of the Oxford bone tumour registry was performed from January 2014 – October 2023. Inclusion criterion was histological confirmation of GCTB. Exclusion criteria were incomplete medical, imaging or histology records, or referral for secondary MDT opinion for diagnosis. From an initial group of 126 GCTB patients, 83 patients met the full selection criteria. Pulmonary metastases were identified in 11 patients. Three with PM were excluded on histopathological review as being giant cell rich osteosarcoma rather than metastatic GCTB. This left 8 (9.6%) patients, one had PM at presentation and seven at follow-up between 2 and 42 months. Two were histologically confirmed after cardiothoracic surgery and biopsy, six radiologically diagnosed. Three (37.5%) patients with PM have died (between 1 and 12 months after confirmed PM), five are alive with stable disease. Seven (87.5%) of patients with pulmonary disease were treated with denosumab/chemotherapy (three before, four after pulmonary diagnosis). Five (62.5%) with pulmonary disease had recurrence of local disease requiring further surgery. Local recurrence was an independent risk factor for PM on statistical analysis. GCTB may present with PM, but more commonly, metastasis occurs after surgery, presenting on surveillance and can progress. There were no distinct differences in histopathological appearance between patients with GCTB that developed PM and those that did not, therefore morphological features of the tumour cannot be currently used to predict tumour behaviour. PM can behave aggressively, necessitating identifying histological markers to recognise patients at risk of metastatic GCTB, for example, through mRNA single cell analysis. We propose GCTB patients with PM receive regular chest surveillance with PET scan and/or CT to monitor disease progression, and a multi-centre audit of GCTB outcome undertaken to further define optimal clinical management.

## Introduction

Giant cell tumour of bone (GCTB) is classified as locally aggressive primary bone tumour [1].

The most common primary tumour sites are meta-epiphyseal regions of long bones, typically the knee joint [2–5]. In the United Kingdom, ≥50 cases of GCTB are diagnosed annually, making up 4%–5% of all primary bone tumours [1, 6]. GCTB has a female to male ratio of between 1.3 and 1.5 to 1, mostly affecting patients aged 20–45. Most present with pain and bone/joint swelling or pathological fracture [7, 8].

Surgery, namely curative resection, is the indicated management [9] and may be in combination with targeted systemic therapy with Denosumab. The indications for denosumab are high risk patients such as those with locally advanced disease, local recurrence, or metastasis. Denosumab has known side effects of arthralgia, fatigue, hypocalcaemia, and rarely osteonecrosis [10, 11]. Surgical treatment varies from curettage and cementoplasty to bone/joint resection and limb reconstruction [12]. Other adjuvant therapies have been used in the past, namely bone grafting, radiotherapy, phenolisation, liquid nitrogen and hydrogen peroxide [13–17].

**FIGURE 1:**
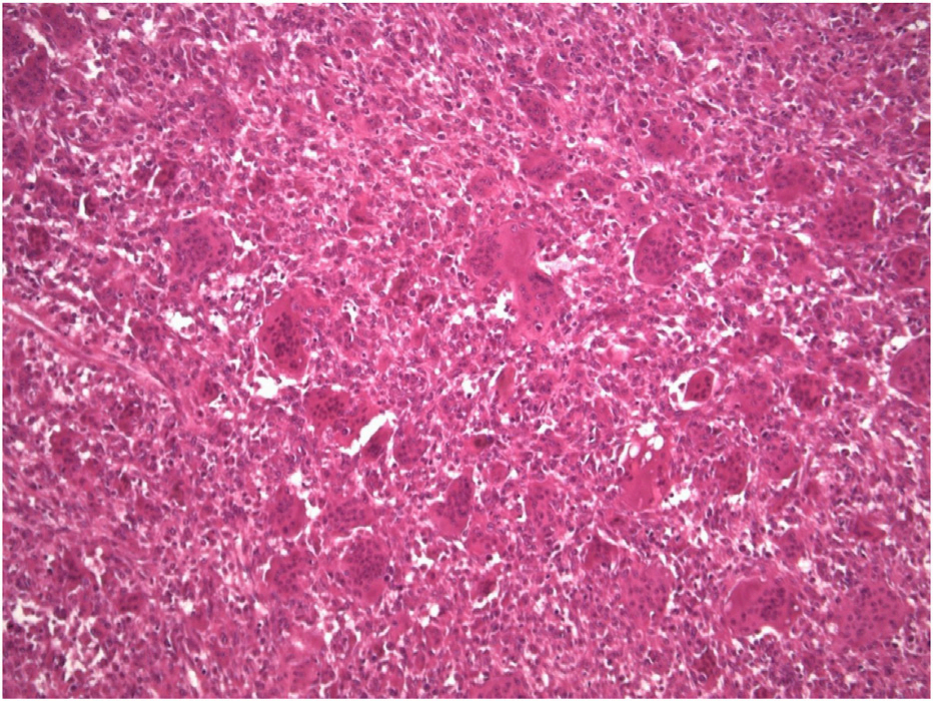
Microscopic appearance of GCTB (Haematoxylin-eosin stain × 10 mag).

**FIGURE 2:**
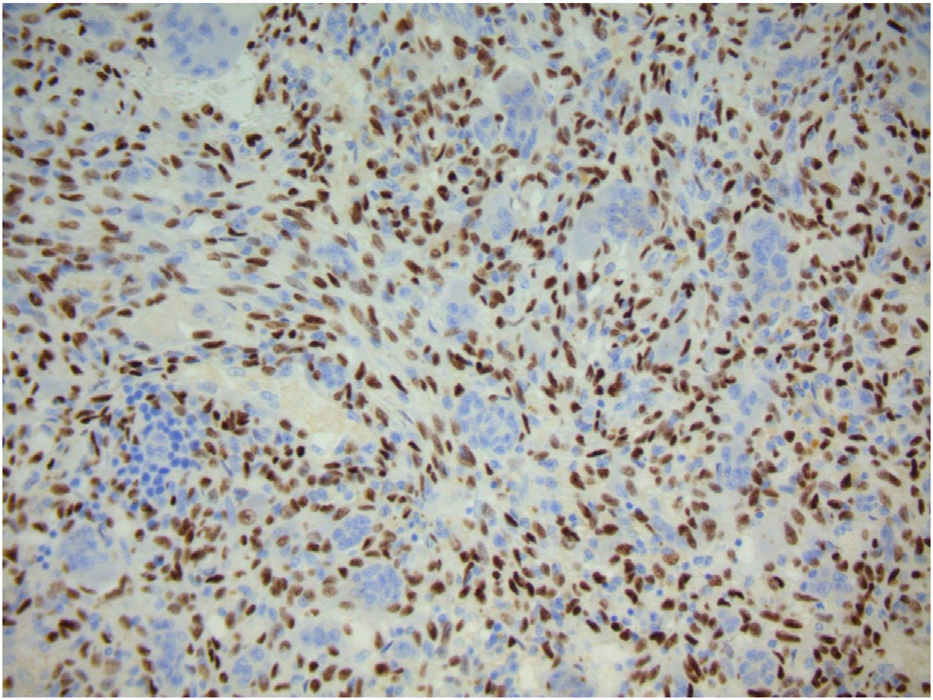
Strong diffuse positive nuclear expression of H3.3G34W immunohistochemical marker by the mononuclear component of giant cell tumour of bone. Giant cells are negative.

Typically, the patient undergoes image guided biopsy for histological diagnosis. Macroscopically, the tumour is haemorrhagic and friable, slightly brownish or red-tan. There may be extensive cortical destruction, and a soft tissue component. Microscopic histological analysis shows a giant cell rich lesion within bone which is composed of three cell types, neoplastic mononuclear stromal cells admixed with macrophages and osteoclast-like giant cells [1, 18, 19] (Figure 1). These three cell types interact with each other via the RANKL-RANK axis and other mechanisms leading to tumour formation. The neoplastic mononuclear stromal cells carry a mutation in the H3F3A gene which, together with the H3F3B gene, encodes the histone protein H3.3 involved in epigenetic regulation of DNA expression. The vast majority of these mutations is a glycine 34 to tryptophan (G34W) substitution [20] with a minor subset (<5%) carrying other H3F3A mutations [21]. The mutated protein is expressed in the nucleus of the neoplastic mononuclear stromal cells, and is highly specific for GCTB (Figure 2). The G34W mutation acts via epigenetic regulatory pathways to modulate secretion of factors, including RANK-ligand, which is expressed by the neoplastic cells. This molecule plays a key role in governing bone metabolism and remodelling and promotes differentiation of osteoclasts resulting in the increased aggressive osteolysis characteristic of GCTB [22]. The discovery of the involvement of the RANK-RANKL signalling pathway has led to treatment of GCTB with RANK inhibitors, such as the human monoclonal antibody Denosumab which binds to RANK and so blocks osteolysis, inhibits tumour growth and helps restore bone density [23].

There is a significant risk of local recurrence (LR) with GCTB, resulting in patients often requiring further surgery with increased morbidity. The incidence of post-surgical LR in GCTB vary from 0%–56% reported (Table 1).

**TABLE 1:** Reported rates of GCTB local recurrence in literature.

Authors and Year	Type of study	Number of patients	Local Recurrence Rate (%)
Zoccali et al 2022 [24]	Systematic review	226	6
Kremen at al 2012 [25]	Retrospective	230	10
Xing et al 2013 [26]	Retrospective	276	11
Saikia et al 2011 [27]	Retrospective	139	11
Luengo-Alonso et al 2019 [28]	Systematic review	1,095	6–12
Gaston et al 2011 [29]	Retrospective	330	12–30
Chanchairujira et al 2007 [30]	Retrospective	74	15
Aoude et al 2023 [31]	Prospective	354	15
Errani et al 2017 [32]	Retrospective	210	16
Balke et al 2008 [15]	Retrospective	214	17
Turcotte et al 2002 [33]	Retrospective	186	17
Becker et al 2024 [34]	Retrospective	643	18
Abuhejleh et al 2020 [35]	Retrospective	57	19
Kito et al 2017 [36]	Retrospective	141	27
Jiang et al 2013 [37]	Retrospective	140	36
Al-Ibraheemi et al 2016 [38]	Retrospective	55	38
Machak et al 2023 [39]	Systematic review	6,441	47
Niu et al 2012 [40]	Retrospective	621	56

Although viewed as a locally aggressive benign tumour, GCTB has metastatic pulmonary potential, [41–43]. GCTB pulmonary metastasis (PM) rates are reported as 0%–8% (Table 2).

**TABLE 2:** Reported rates of metastatic disease in literature.

Authors and Year	Type of study	Number of patients	PM rate (%)
Abuhejleh et al 2020 [35]	Prospective	57	0
Zoccali et al 2022 [24]	Systematic review	226	0.9
Kremen et al 2012 [25]	Retrospective	230	2
Dominkus 2006 [44]	Retrospective	649	2.1
Xing et al 2013 [26]	Retrospective	276	2.2
Lans et al 2020 [45]	Retrospective	82	2.4
Niu et al 2012 [40]	Retrospective	621	3.4
Al-Ibraheemi et al 2016 [38]	Retrospective	55	3.6
Viswanathan et al 2010 [46]	Retrospective	470	4.5
Kamal et al 2016 [47]	Retrospective	82	4.9
Tsakamoto et al 2019 [48]	Retrospective	381	5
Becker 2024 [34]	Retrospective	643	5.1
Wang et al 2021 [49]	Retrospective	310	5.8
Yayan 2019 [50]	Systematic review	4,295	5.1–6.5
Chan et al 2015 [51]	Retrospective	167	6.6
Luengo-Alonso et al 2019 [28]	Systematic review	1,095	1.0–7.0
Rosario et al 2017 [52]	Prospective	333	7.5
Jiang et al 2013 [37]	Retrospective	140	7.9
Kito et al 2017 [36]	Retrospective	141	8.5

Rarely, the tumour can undergo a malignant transformation and is classified as either primary or more commonly secondary malignant GCTBs, the latter as a result of radiotherapy [43, 53–57].

Metastatic disease is viewed as having a benign course [58, 59], however, is associated with higher mortality [36, 47, 60]. With PM, there is risk of progressive respiratory disease and death [37, 50]. As such, recognition and monitoring of PM through standardised surveillance is essential. Management of PM requires surveillance for cardiothoracic surgical management with/without neoadjuvant therapy [43, 54, 61].

To identify occult and metastatic pulmonary disease, patients are routinely followed up with surveillance scanning of extremity and thoracic imaging with PET/CT [36, 52, 62, 63].

LR is known to be an independent risk factor for PM, with other known risk factors namely, primary tumour site, patient age, Campanacci grade, modality of surgical treatment, and local site radiation [36, 48–52].

The primary aim of this study was to determine the true incidence of PM and current surveillance protocols. This would then be used to create recommendations on national surveillance protocols for this unpredictable disease.

## Methods

A retrospective audit looking at GCTB patient outcomes identified from the Oxford Sarcoma Registry was performed. The study was registered in the Oxford University Hospitals audit system, receiving ethical approval from the local research ethics committee, reference number 7,605. The study was preformed in accordance with the ethical standards as described in the 1964 Declaration of Helsinki. All patients were diagnosed and treated at Oxford University Hospitals NHS Foundation Trust, and consent was obtained for treatment. As part of the Oxford University Hospitals consent process, all patients consented to their data being used for research and publication purposes. All patient data was anonymised.

Patient records were searched between January 2014 to October 2023. 170 histopathology records were identified, of which 126 were individual patients. The inclusion criterion was histological confirmation of primary GTCB. Exclusion criteria were incomplete medical, imaging or pathology records, referral for a secondary histopathological multidisciplinary team opinion for diagnosis, and histopathological diagnosis of pathology other than primary giant cell tumour of bone on repeat histology.

**FIGURE 3:**
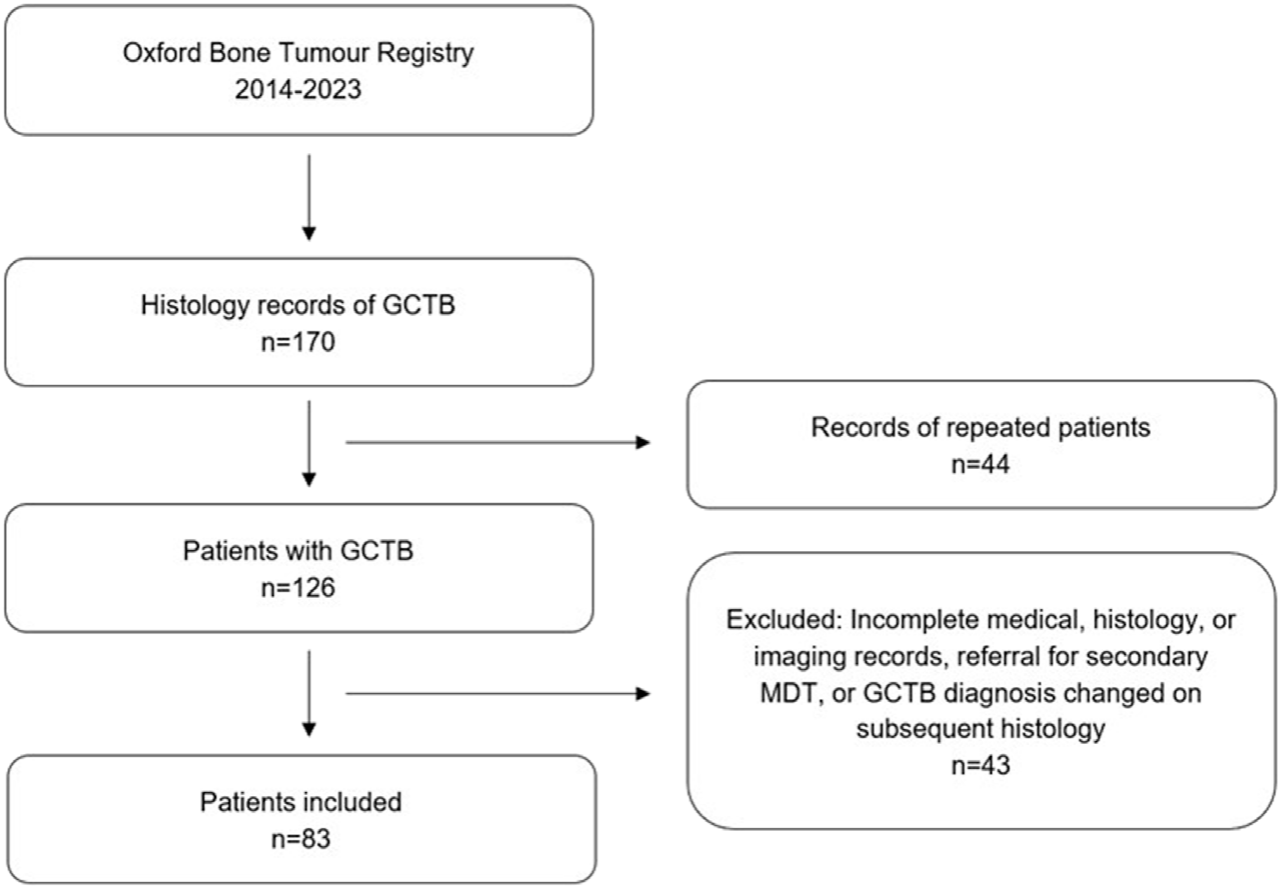
Flowchart showing study design including inclusion/exclusion criteria.

Of note, three cases with PM were excluded, as they originally showed histological features of GCTB, but diagnosis was changed to osteosarcoma giant cell variant on subsequent sample histopathological review. 43 patients were excluded, leaving 83 for analysis (Figure 3).

The clinical data collected included patient demographics, correlated radiopathology imaging, detailed panelled histology, site of primary tumour, type of surgical and systemic treatment, and LR.

Criteria for diagnosis of PM were either a histological confirmation or enlarging pulmonary nodules on at least two consecutive dedicated CT scans.

Metastatic surveillance protocols were collated from United Kingdom and international bone tumour centres for comparison of surveillance for PM. Birmingham United Kingdom, Newcastle United Kingdom, Oswestry United Kingdom, Aberdeen United Kingdom, Glasgow United Kingdom, Leiden Netherlands, and Perth Australia were asked their current local protocols for surveillance of GCTB.

### Statistical analysis

Patient and tumour variables which included gender, age, location of primary tumour, soft tissue invasion of primary tumour, pathological fracture from primary tumour, denosumab therapy prior to diagnosis of PM if applicable, type of surgery, and LR, were collected and analysed as possible risk factors for PM using univariate and multivariate logistic regression statistical analysis in R 4.3.1.

## Results

Mean patient age at presentation was 36.4 (range 15–81). Mean follow up time was 44.0 months (range 0–130).

All patients were discussed at regional bone tumour MDT for recommendation of treatment. 77 (93.9%) patients were treated surgically, one treated non operatively with denosumab to good effect, two deemed unfit for surgery and died within 1 year of presentation, and three were being treated with neoadjuvant denosumab at time of data collection. Primary surgical treatment included curettage, excision with reconstruction, or excision followed by joint arthroplasty. Surgical treatment of recurrent disease included the listed options and amputation. Considerations for choice of treatment included location of primary disease, radiological appearance, periosteal and/or soft tissue invasion, and options for surgical reconstruction.

Metastatic disease occurred in eight (9.6%) patients and all metastases were pulmonary. One patient had metastatic disease at diagnosis, seven were identified at follow-up between 2 and 42 months (mean = 20.6) after presentation. Of the eight patients with PM, two were confirmed histologically after one underwent surgery for metastasectomy and one had biopsy, six diagnosed through CT/PET imaging (Table 3).

**TABLE 3:** Summary of characteristics of patient demographics, pulmonary metastases, primary tumour, primary surgical treatment, and denosumab treatment.

Characteristic	Number/n	Frequency/%
Patient Demographics		
Total	83	100
Men	43	51.8
Women	40	48.2
Age at diagnosis >40	26	31.3
Age at diagnosis ≤40	57	68.7
Pulmonary Metastases		
At presentation	1	1.2
At follow up	7	8.4
None	75	90.4
Primary Tumour		
Lower limb	51	61.4
Upper Limb	17	20.5
Axial skeleton	15	18.1
Pathological fracture	22	26.5
Soft tissue invasion	18	21.7
Local Recurrence	18	21.7
Primary Surgical Treatment		
Curettage	46	55.4
Other	31	37.3
No surgery	6	7.2
Denosumab Treatment		
Neoadjuvant	22	26.5
Adjuvant	14	16.9
None	47	56.6

Of the two diagnosed histologically, the one diagnosed on biopsy showed partial fibrosis and relatively large number of giant cells on histology review, which could indicate effect to denosumab treatment. The one treated with metastasectomy showed a more convincing denosumab treatment effect in the form of fibrosis and bone formation, and Giant cells had disappeared.

Of the patients with PM, three (37.5%) died (between 6 and 12 months after confirmed PM), five alive with stable disease. 35 (42.2%) of all 83 patients were treated with denosumab as per MDT recommendation. Seven patients (87.5%) with PM were treated with denosumab (three before, four after pulmonary diagnosis) (Table 4). No patients with PM had radiotherapy.

**TABLE 4:** Results from the 8 patients with metastatic chest disease. Primary tumour site, time to pulmonary disease from GCTB diagnosis, local recurrence free survival, whether they had systemic treatment, survival after pulmonary disease, gender, and age at GCTB diagnosis. DX, diagnosis; Tx, treatment, pul. disease, pulmonary disease.

Case	Primary tumour site	Time to pul. disease after GCTB Dx	Local recurrence free survival	Systemic Tx before/after pul. disease	Survival after pul. disease	Gender	Age at Dx
1	L1 vertebra	At presentation	No surgery	After	6 months	Male	35
2	Proximal femur	2 months	No recurrence	After	Alive	Male	21
3	Distal femur	9 months	9 months	After	9 months	Male	68
4	Metacarpal	16 months	No recurrence	No systemic treatment	Alive	Male	40
5	Middle finger	20 months	15 months	Before	12 months	Female	16
6	Patella	22 months	4 months	Before	Alive	Male	33
7	Calcaneum	33 months	32 months	After	Alive	Male	32
8	Proximal tibia	42 months	6 months	Before	Alive	Female	22

18 patients (21.7%) had LR, of which 16 (88.9%) were treated with denosumab (nine treated before diagnosis of recurrence and seven after). Five (62.5%) of the eight patients with PM had LR and all required surgery of recurrence.

One patient from all 83 in the study had primary malignant GCTB at time of diagnosis, and that patient developed PM.

Statistical analysis showed that LR was the only significant risk factor for PM, on both univariate and multivariate logistic regression analysis (Table 5).

**TABLE 5:** Statistical analysis of patient variables looking at risk factors for chest disease.

Univariate			
Variable	Odds ratio	95% confidence interval	p value
Male sex	3.08	0.66–22.0	0.19
Age at diagnosis ≤40	3.50	0.58–63.3	0.25
Location of primary in lower limb	1.05	0.24–5.43	0.95
Surgery type (curettage vs. other)	2.90	0.35–12.9	0.51
Local recurrence	11.0	2.11–82.8	^a^0.007
Pathological fracture at diagnosis	0.92	0.13–4.37	0.92
Denosumab therapy	1.31	0.30–6.76	0.73
Soft tissue invasion	4.36	0.93–20.6	0.055
Multivariate			
Male sex	4.49	0.60–55.1	0.18
Age at diagnosis ≤40	4.54	0.27–430	0.40
location of primary in lower limb	0.48	0.05–4.99	0.51
Surgery type (curettage vs. other)	1.70	0.26–45.7	0.44
Local recurrence	67.73	5.14–10,777	^a^0.013
Pathological fracture at diagnosis	1.45	0.19–11.1	0.48
Denosumab therapy	11.58	0.67–743	0.15
Soft tissue invasion	2.09	0.31–41.6	0.33

^a^ Statistically significant (p < 0.05).

Differences between surveillance protocols across specialist sarcoma centres were found. All follow-up protocols are between 5 and 10 years (Table 6).

**TABLE 6:** GCTB follow-up and surveillance protocols from different sarcoma centres.

Centre	Chest surveillance protocol
Oxford, United Kingdom	Baseline CT chest or PET-CT at diagnosis 3 monthly chest x-ray up to 2 years, 6 monthly from 2–5 years, annually from 5–10 years If chest disease found, CT chest/PET CT and referral to cardiothoracic surgeons to see if resectable
Birmingham, United Kingdom	Chest x-ray on diagnosis, no other chest imaging unless local recurrence
Newcastle, United Kingdom	Chest x-ray at diagnosis, then annually for surveillance
Oswestry, United Kingdom	Chest x-ray at diagnosis, then annually for surveillance. CT chest considered if local recurrence
Aberdeen, United Kingdom	Chest x-ray at diagnosis, surveillance guided by aggressive of disease on pathology and radiology
Glasgow, United Kingdom	Chest x-ray on diagnosis, no other chest imaging unless local recurrence
Leiden, Netherlands	At diagnosis, since 2010. 2 yearly chest imaging with XR
Perth, Australia	Baseline CT chest at diagnosis. Follow-up imaging 6 monthly for 2 years, then annually for 2 years. Total follow-up 4 years. If chest disease found, aim to resect is possible, followed by 4 monthly scans for 2 years, 6 monthly for next 2 years, then annual until 8 years total

## Discussion

GCTB is an unpredictable disease and whilst most cases have a clinically benign course, there is a risk of progressive and latent PM. The results of this study demonstrate PM rate of 9.6%, suggesting that metastasis rate of GCTB to the lungs is higher than reported from historical data. This may be explained partly by the advancements in 3-D imaging, either thin section CT or PET scan, which can identify small volume disease not evident on standard chest x-ray. The risk of latent and progressive disease is a risk of aggressive and fatal PM, so would necessitate CT/PET at presentation and follow-up.

Treatment options for PM include observation and symptomatic treatment, metastasectomy, denosumab, chemotherapy, and radiotherapy. The decision of treatment options is complex and based off MDT discussion, taking into account patient fitness for surgery or systemic treatment, and aggressiveness of disease [43, 46].

62.5% of patients with PM had LR, and analysis showed LR was a statistically significant risk factor for PM, in keeping with current literature.

Therefore, when LR is found, as there is restaging of limb recurrence with MRI, the chest would need careful assessment with CT and/or PET rather than chest x-ray imaging.

Further assessment of the statistical analysis shows that the other patient and tumour variables tested were not statistically significant risk factors for PM, and some of the 95% confidence intervals were very wide, more so on multivariate analysis. This is likely to be due to the relatively small data set.

**FIGURE 4:**
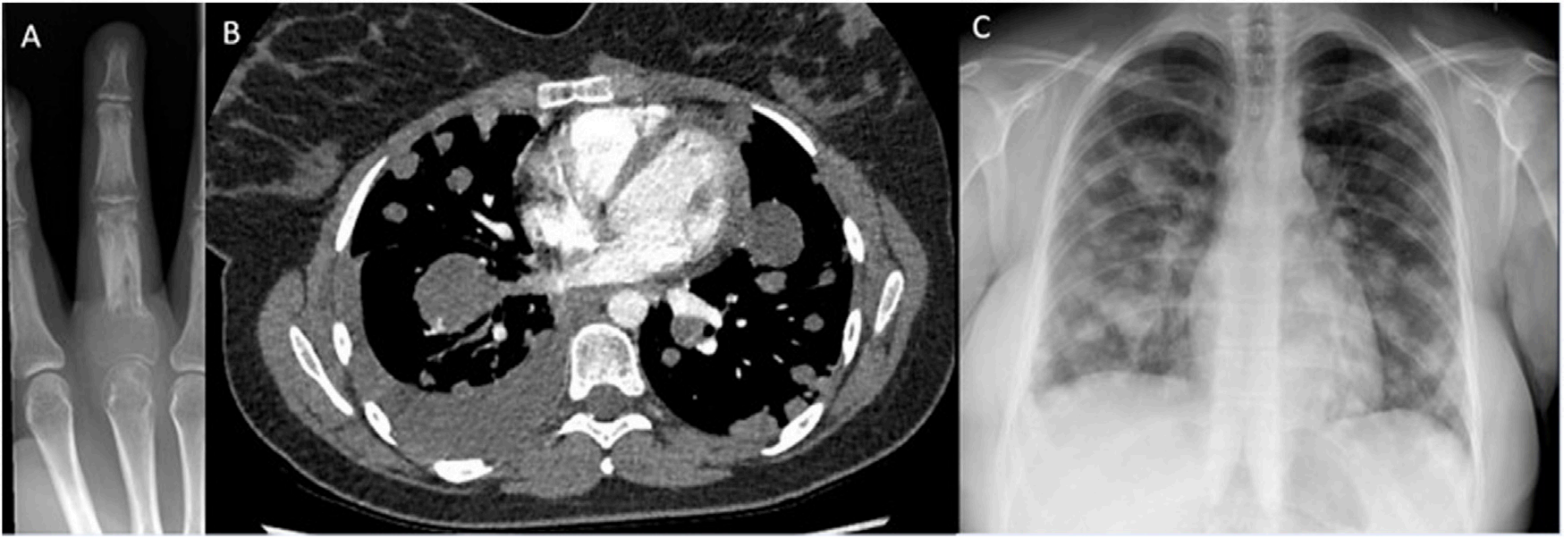
Imaging of GCTB from one of the patients. **(A)** – X-ray right middle finger 24/09/2018, of primary GCTB tumour. **(B)** – CT chest, soft tissue window 13/05/2020, showing pulmonary metastatic spread, **(C)** – X-ray chest 24/08/2020, showing metastatic spread.

In this review, 37.5% of patients with PM have died within 12 months of radiological diagnosis of PM, showing that when GCTB does metastasise, it is often unstable and carries a high morbidity and mortality rate. Figure 4 shows imaging of a patient with small volume primary GCTB of the proximal phalanx with secondary aggressive PM found on x-ray and staging CT.

Histologically with GCTB it is difficult to predict the risk of metastatic disease. In terms of the histological diagnosis of GCTB, the mutant histone protein H3.3G34W can now be reliably detected in the neoplastic stromal cell population by immunohistochemistry and it serves as a highly specific surrogate marker for this tumour [64]. Expression of the mutant protein is not detected in osteoclasts or their precursors, or by other giant cell rich lesions that mimic GCTB [64]. This marker is often preserved with malignant transformation. In those cases where it is absent, it is proposed that the H3F3AG34W mutation is lost during clonal evolution of the tumour [65].

Three patients initially included, were subsequently excluded when review of their histology changed diagnosis from GCTB to metastatic osteosarcoma. These were tested for H3.3G34W through immunochemistry. Two of these were negative for H3.3G34W, one was positive for the marker. Osteosarcoma can be positive for H3.3G34W in 2.85% of cases [66] and there was no residual benign GCTB areas on histopathological review in this case.

Although the much rarer, primary and secondary malignant giant cell tumours show clear morphological and gene expression correlates reflecting sarcomatous transformation it has proved difficult to pinpoint histological markers that may indicate the metastatic potential of clinically benign cases of GCTB. Morphologically, malignant GCTB have an admixed sarcomatous component, decreased numbers of osteoclast-like giant cells and overt nuclear atypia in neoplastic stromal cells as well as multinucleated giant cells. Gong et al [60] showed in cases of primary and secondary malignant GCTB that expression of p53 and the proliferation marker Ki-67 is increased. Other studies have identified a subset of giant cell tumours which express high levels of beta-HCG, likely a para-neoplastic phenomenon, and it has been suggested markedly elevated beta-hCG expression and secretion may carry a worse prognosis [64].

In contrast, prognostic histological markers for clinically benign GCTB have proved elusive and it remains difficult to predict the behaviour of these tumours at presentation. Antal et al described a technique using smear cytophotometry and proliferation activity by Ki-67 MIB immunohistochemistry to assess DNA ploidy as a possible prognostic marker [67]. Although it has been reported that Ki-67 levels can increase during repeated recurrences [68], studies have not found a significance difference in Ki67, p53, p63, cyclin D1 or Bcl-2 expression between patients who develop PM and LR and those that did not [69, 70]. However, recent molecular studies are more encouraging [71], and multiplex gene analysis methods have suggested that MDM2, IGF1, STAT1 and the GTPase family member RAC1 may be associated with GCTB recurrence [72], raising the possibility that these could be used as markers in the future. Furthermore, gene expression studies show increased LR rates for GCTB are associated with higher levels of expression of the immunomodulatory gene PDL-1 and altered expression of a subset of immuno-system related genes [73] and this may be an area to explore further in identifying prognostic factors for this unpredictable tumour.

Pulmonary metastases require close monitoring with PET and CT scanning and MDT-led treatment decision on metastasectomy surgery with considered adjuvant systemic therapy.

Main limitations to this study include data collection from a single centre and a relatively small data set. However, this has been performed at a specialised unit with experience in managing this unpredictable primary bone tumour. To further validate the data presented from this single unit study, it would further require a multi-centre study of surveillance of GCTB and PM disease.

There is currently no national, or international consensus on surveillance of GCTB, as shown by the variations in protocols between the sarcoma centres described in Table 5. It has been previously suggested that GCTB warrants strict follow-up due to the risk of GCTB malignant transformation and metastatic spread which although rare, carries significant morbidity and mortality.

We would recommend baseline CT chest or PET-CT at diagnosis, with a follow-up CT chest 6 months after surgery or if there is evidence of LR at primary site. Then three monthly chest x-ray up to 2 years, six monthly from 2–5 years, annually from 5–10 years. If PM found, CT chest/PET and MDT review with cardiothoracics for management of resectable disease. We would recommend a national collaboration for a surveillance protocol.

## Conclusion

High incidence of PM of >9% was observed in this study, which is higher than reported historically. This result suggests that more rigorous chest surveillance is required with CT chest and/or PET CT at diagnosis and at six-month follow-up with surveillance for 5 years for PM and LR which notably remains a significant risk factor for PM. Further steps are needed to identify markers for malignant transformation potential.

## Data Availability

The original contributions presented in the study are included in the article/supplementary material, further inquiries can be directed to the corresponding author.

## References

[1] Siegal GPHM Bloem JL Cates JMM. WHO classification of tumors 5th edition soft tissue and bone tumors. 5th ed. Geneva, Switzerland: World Health Organization (2020). 440–7.

[2] Turcotte RE. Giant cell tumor of bone. Orthop Clin North Am (2006) 37(1):35–51. 10.1016/j.ocl.2005.08.005 16311110

[3] Unni KK Inwards CY. Dahlin’s bone tumors: general aspects and data on 10,165 cases. 6th ed. Philadelphia, United States: Lippincott Williams and Wilkins (2012).

[4] Yang Y Huang Z Niu X Xu H Li Y Liu W. Clinical characteristics and risk factors analysis of lung metastasis from benign giant cell tumor of bone. J Bone Oncol (2017)(7) 23–8. 10.1016/j.jbo.2017.04.001 PMC5397572 28443231

[5] Barik S Jain A Ahmad S Singh V. Functional outcome in giant cell tumor of distal radius treated with excision and fibular arthroplasty: a case series. Eur J Orthop Surg Traumatol (2020) 30(6):1109–17. 10.1007/s00590-020-02679-2 32358713

[6] Sarcoma. Giant cell tumour of the bone. Available from: https://sarcoma.org.uk/about-sarcoma/what-is-sarcoma/types-of-sarcoma/giant-cell-tumour-of-the-bone/ (Accessed November 30, 2023).

[7] Hosseinzadeh S De Jesus O. Giant cell tumor. StatPearls. Treasure Island (FL): StatPearls Publishing (2023). Available from: https://www.ncbi.nlm.nih.gov/books/NBK559229/ (Accessed January 8, 2024). 32644655

[8] Kundu ZS Sen R Dhiman A Sharma P Siwach R Rana P. Effect of intravenous zoledronic acid on histopathology and recurrence after extended curettage in giant cell tumors of bone: a comparative prospective study. Indian J Orthop (2018) 52(1):45–50. 10.4103/ortho.IJOrtho_216_17 29416169 PMC5791231

[9] Akiyama T Yoshimatsu Y Noguchi R Sin Y Tsuchiya R Ono T Establishment and characterization of NCC-GCTB5-C1: a novel cell line of giant cell tumor of bone. Hum Cel (2022) 35(5):1621–9. 10.1007/s13577-022-00724-2 35653034

[10] Brodowicz T Hemetsberger M Windhager R. Denosumab for the treatment of giant cell tumour of the bone. Future Oncol (2015) 11(13):1881–94. 10.2217/fon.15.94 26161925

[11] Imre A Zoltan S Miklos S. Current indications for denosumab in benign bone tumours. EFFORT Open Rev (2023) 8(12):895–905. 10.1530/EOR-23-0138 PMC10714381 38038377

[12] van der Heijden L Dijkstra PDS van de Sande MAJ Kroep JR Nout RA van Rijswijk CSP The clinical approach toward giant cell tumor of bone. The Oncologist (2014) 19(5):550–61. 10.1634/theoncologist.2013-0432 24718514 PMC4012970

[13] Caudell JJ Ballo MT Zagars GK Lewis VO Weber KL Lin PP Radiotherapy in the management of giant cell tumor of bone. Int J Radiat Oncol Biol Phys (2003) 57(1):158–65. 10.1016/s0360-3016(03)00416-4 12909228

[14] Errani C Tsukamoto S Ciani G Donati DM. Present day controversies and consensus in curettage for giant cell tumor of bone. J Clin Orthop Trauma (2019) 10(6):1015–20. 10.1016/j.jcot.2019.09.017 31736607 PMC6844202

[15] Balke M Schremper L Gebert C Ahrens H Streitbuerger A Koehler G Giant cell tumor of bone: treatment and outcome of 214 cases. J Cancer Res Clin Oncol (2008) 134(9):969–78. 10.1007/s00432-008-0370-x 18322700 PMC12160765

[16] Arbeitsgemeinschaft K Becker WT Dohle J Bernd L Braun A Cserhati M Local recurrence of giant cell tumor of bone after intralesional treatment with and without adjuvant therapy. J Bone Joint Surg Am (2008) 90(5):1060–7. 10.2106/JBJS.D.02771 18451399

[17] van der Heijden L Dijkstra PDS Blay JY Gelderblom H. Giant cell tumour of bone in the denosumab era. Eur J Cancer (2017) 77:75–83. 10.1016/j.ejca.2017.02.021 28365529

[18] Atkins GJ Haynes DR Graves SE Evdokiou A Hay S Bouralexis S Expression of osteoclast differentiation signals by stromal elements of giant cell tumors. J Bone Miner Res Off J Am Soc Bone Miner Res (2000) 15(4):640–9. 10.1359/jbmr.2000.15.4.640 10780856

[19] Wülling M Delling G Kaiser E. The origin of the neoplastic stromal cell in giant cell tumor of bone. Hum Pathol (2003) 34(10):983–93. 10.1053/s0046-8177(03)00413-1 14608531

[20] Behjati S Tarpey PS Presneau N Scheipl S Pillay N Van Loo P Distinct H3F3A and H3F3B driver mutations define chondroblastoma and giant cell tumor of bone. Nat Genet (2013) 45(12):1479–82. 10.1038/ng.2814 24162739 PMC3839851

[21] Yamamoto H Ishihara S Toda Y Oda Y. Histone H3.3 mutation in giant cell tumor of bone: an update in pathology. Med Mol Morphol (2020) 53(1):1–6. 10.1007/s00795-019-00238-1 31748824

[22] Lutsik P Baude A Mancarella D Öz S Kühn A Toth R Globally altered epigenetic landscape and delayed osteogenic differentiation in H3.3-G34W-mutant giant cell tumor of bone. Nat Commun (2020) 11(1):5414. 10.1038/s41467-020-18955-y 33110075 PMC7591516

[23] Chawla S Blay JY Rutkowski P Le Cesne A Reichardt P Gelderblom H Denosumab in patients with giant-cell tumour of bone: a multicentre, open-label, phase 2 study. Lancet Oncol (2019) 20(12):1719–29. 10.1016/S1470-2045(19)30663-1 31704134

[24] Zoccali C Formica VM Sperduti I Checcucci E Scotto di Uccio A Pagnotta A Wide resection for giant-cell tumor of the distal radius: which reconstruction? A systematic review of the literature and pooled analysis of 176 cases. Hand Surg Rehabil (2022) 41(5):552–60. 10.1016/j.hansur.2022.07.002 35868588

[25] Kremen TJ Jr Bernthal NM Eckardt MA Eckardt JJ. Giant cell tumor of bone: are we stratifying results appropriately? Clin Orthop Relat Res (2012) 470(3):677–83. 10.1007/s11999-011-2172-8 22125240 PMC3270191

[26] Xing R Yang J Kong Q Tu C Zhou Y Duan H. Giant cell tumour of bone in the appendicular skeleton: an analysis of 276 cases. Acta Orthop Belg (2013) 79(6):731–7. 10.1302/2058-5241.6.200154 24563982

[27] Saikia KC Bhuyan SK Borgohain M Saikia SP Bora A Ahmed F. Giant cell tumour of bone: an analysis of 139 Indian patients. J Orthop Sci (2011) 16(5):581–8. 10.1007/s00776-011-0033-7 21833611

[28] Luengo-Alonso G Mellado-Romero M Shemesh S Ramos-Pascua L Pretell-Mazzini J. Denosumab treatment for giant-cell tumor of bone: a systematic review of the literature. Arch Orthop Trauma Surg (2019) 139(10):1339–49. 10.1007/s00402-019-03167-x 30877429

[29] Gaston CL Bhumbra R Watanuki M Abudu AT Carter SR Jeys LM Does the addition of cement improve the rate of local recurrence after curettage of giant cell tumours in bone? J Bone Joint Surg Br (2011) 93(12):1665–9. 10.1302/0301-620X.93B12.27663 22161931

[30] Chanchairujira K Jiranantanakorn T Phimolsarnti R Asavamongkolkul A Waikakul S. Factors of local recurrence of giant cell tumor of long bone after treatment: plain radiographs, pathology and surgical procedures. J Med Assoc Thai (2011) 94(10):1230–7. 22145509

[31] Aoude A Nikomarov D Perera JR Ibe IK Griffin AM Tsoi KM Giant cell tumour of bone. Bone Joint J (2023) 105-B(5):559–67. 10.1302/0301-620X.105B5.BJJ-2022-1231.R1 37121582

[32] Errani C Tsukamoto S Leone G Akahane M Cevolani L Tanzi P Higher local recurrence rates after intralesional surgery for giant cell tumor of the proximal femur compared to other sites. Eur J Orthop Surg Traumatol (2017) 27(6):813–9. 10.1007/s00590-017-1983-z 28589498

[33] Turcotte RE Wunder JS Isler MH Bell RS Schachar N Masri BA Giant cell tumor of long bone: a Canadian Sarcoma Group study. Clin Orthop Relat Res (2002) 397:248–58. 10.1097/00003086-200204000-00029 11953616

[34] Becker RG Galia CR Pestilho JFCS Antunes BP Baptista AM Guedes A. Giant cell tumor of bone: a multicenter epidemiological study in Brazil. Acta Ortop Bras (2024) 32(1):e273066. 10.1590/1413-785220243201e273066 38532872 PMC10962070

[35] Abuhejleh H Wunder JS Ferguson PC Isler MH Mottard S Werier JA Extended intralesional curettage preferred over resection-arthrodesis for giant cell tumour of the distal radius. Eur J Orthop Surg Traumatol Orthop Traumatol (2020) 30(1):11–7. 10.1007/s00590-019-02496-2 31297594

[36] Kito M Matusmoto S Ae K Tanizawa T Gokita T Kobayashi H Pulmonary metastasis from giant cell tumor of bone: clinical outcome prior to the introduction of molecular target therapy. Jpn J Clin Oncol (2017) 47(6):529–34. 10.1093/jjco/hyx033 28334868

[37] Jiang N Qin CH Tan CX Wen SF Ma YF Dong F A retrospective analysis of 140 patients with giant cell tumor in the extremity: a multicenter study based on four hospitals in South China. Cancer Epidemiol (2013) 37(3):294–9. 10.1016/j.canep.2013.01.009 23419818

[38] Al-Ibraheemi A Inwards CY Zreik RT Wenger DE Jenkins SM Carter JM Histologic spectrum of giant cell tumor (GCT) of bone in patients 18 Years of age and below: a study of 63 patients. Am J Surg Pathol (2016) 40(12):1702–12. 10.1097/PAS.0000000000000715 27526293

[39] Machak GN Snetkov AI. The impact of curettage technique on local control in giant cell tumour of bone. Int Orthop (2021) 45(3):779–89. 10.1007/s00264-020-04860-y 33094400

[40] Niu X Zhang Q Hao L Ding Y Li Y Xu H Giant cell tumor of the extremity: retrospective analysis of 621 Chinese patients from one institution. JBJS (2012) 94(5):461–7. 10.2106/JBJS.J.01922 22398741

[41] Vari S Riva F Onesti CE Cosimati A Renna D Biagini R Malignant transformation of giant cell tumour of bone: a review of literature and the experience of a referral centre. Int J Mol Sci (2022) 23(18):10721. 10.3390/ijms231810721 36142631 PMC9506170

[42] Leland CR Pratilas CA Gross JM Levin AS. Diffuse pulmonary metastases at presentation of giant cell tumor of bone: a case report and synthesis of literature. JBJS Case Connect (2023) 13(1). 10.2106/JBJS.CC.22.00496 36821126

[43] Tsukamoto S Mavrogenis AF Tanaka Y Kido A Honoki K Tanaka Y Metastasectomy versus non-metastasectomy for giant cell tumor of bone lung metastases. Orthopedics (2021) 44(6):e707–e712. 10.3928/01477447-20211001-01 34618641

[44] Dominkus M Ruggieri P Bertoni F Briccoli A Picci P Rocca M Histologically verified lung metastases in benign giant cell tumours--14 cases from a single institution. Int Orthop (2006) 30(6):499–504. 10.1007/s00264-006-0204-x 16909252 PMC3172731

[45] Lans J Oflazoglu K Lee H Harness NG Castelein RM Chen NC Giant cell tumors of the upper extremity: predictors of recurrence. J Hand Surg (2020) 45(8):738–45. 10.1016/j.jhsa.2020.04.020 32616409

[46] Viswanathan S Jambhekar NA. Metastatic giant cell tumor of bone: are there associated factors and best treatment modalities? Clin Orthop Relat Res (2010) 468(3):827–33. 10.1007/s11999-009-0966-8 19597900 PMC2816751

[47] Kamal AF Simbolon EL Prabowo Y Hutagalung EU. Wide resection versus curettage with adjuvant therapy for giant cell tumour of bone. J Orthop Surg Hong Kong (2016) 24(2):228–31. 10.1177/1602400221 27574268

[48] Tsukamoto S Mavrogenis AF Leone G Righi A Akahane M Tanzi P Denosumab does not decrease the risk of lung metastases from bone giant cell tumour. Int Orthop (2019) 43(2):483–9. 10.1007/s00264-018-4085-6 30099641

[49] Wang J Liu X Yang Y Yang R Tang X Yan T Pulmonary metastasis of giant cell tumour: a retrospective study of three hundred and ten cases. Int Orthop (2021) 45(3):769–78. 10.1007/s00264-020-04907-0 33427899

[50] Yayan J. Increased risk of lung metastases in patients with giant cell bone tumors: a systematic review. Adv Exp Med Biol (2019) 1176:1–17. 10.1007/5584_2019_372 30989587

[51] Chan CM Adler Z Reith JD Gibbs CP Jr. Risk factors for pulmonary metastases from giant cell tumor of bone. J Bone Joint Surg Am (2015) 97(5):420–8. 10.2106/JBJS.N.00678 25740033

[52] Rosario M Kim HS Yun JY Han I. Surveillance for lung metastasis from giant cell tumor of bone. J Surg Oncol (2017) 116(7):907–13. 10.1002/jso.24739 28650536

[53] Fittall MW Lyskjaer I Ellery P Lombard P Ijaz J Strobl AC Drivers underpinning the malignant transformation of giant cell tumour of bone. J Pathol (2020) 252(4):433–40. 10.1002/path.5537 32866294 PMC8432151

[54] Tsukamoto S Righi A Vanel D Honoki K Donati DM Errani C. Development of high-grade osteosarcoma in a patient with recurrent giant cell tumor of the ischium while receiving treatment with denosumab. Jpn J Clin Oncol (2017) 47(11):1090–6. 10.1093/jjco/hyx112 29048579

[55] Park A Cipriano CA Hill K Kyriakos M McDonald DJ. Malignant transformation of a giant cell tumor of bone treated with denosumab: a case report. Jbjs Case Connect (2016) 6(3):e78. 10.2106/JBJS.CC.16.00024 29252655

[56] Bertoni F Bacchini P Staals EL. Malignancy in giant cell tumor of bone. Cancer (2003) 97(10):2520–9. 10.1002/cncr.11359 12733152

[57] Liu W Chan CM Gong L Bui MM Han G Letson GD Malignancy in giant cell tumor of bone in the extremities. J Bone Oncol (2021) 26:100334. 10.1016/j.jbo.2020.100334 33251099 PMC7680773

[58] Chakarun CJ Forrester DM Gottsegen CJ Patel DB White EA Matcuk GR Jr. Giant cell tumor of bone: review, mimics, and new developments in treatment. Radiographics (2013) 33(1):197–211. 10.1148/rg.331125089 23322837

[59] Yeo CD Roh SY Shin OR Bahk WJ Kim KH Kim JW. A case of pulmonary metastasis of giant cell tumor of bone presenting as pulmonary arteriovenous malformation. J Formos Med Assoc (2015) 114(4):369–72. 10.1016/j.jfma.2012.03.014 25839772

[60] Gong L Liu W Sun X Sajdik C Tian X Niu X Histological and clinical characteristics of malignant giant cell tumor of bone. Virchows Arch (2012) 460(3):327–34. 10.1007/s00428-012-1198-y 22350004

[61] Muheremu A Niu X. Pulmonary metastasis of giant cell tumor of bones. World J Surg Oncol (2014) 12:261. 10.1186/1477-7819-12-261 25139054 PMC4155080

[62] Xu R Choong PFM. Metastatic giant cell tumour of bone: a narrative review of management options and approaches. ANZ J Surg (2022) 92(4):691–6. 10.1111/ans.17520 35143093 PMC9303226

[63] van Langevelde K McCarthy CL. Radiological findings of denosumab treatment for giant cell tumours of bone. Skeletal Radiol (2020) 49(9):1345–58. 10.1007/s00256-020-03449-1 32335707 PMC7360539

[64] Lawless ME Jour G Hoch BL Rendi MH. Beta-human chorionic gonadotropin expression in recurrent and metastatic giant cell tumors of bone: a potential mimicker of germ cell tumor. Int J Surg Pathol (2014) 22(7):617–22. 10.1177/1066896914534466 24831855

[65] Yoshida K Nakano Y Honda-Kitahara M Wake S Motoi OK Absence of H3F3A mutation in a subset of malignant giant cell tumour of bone. Mod Pathol (2019) 32(12):1751–61. 10.1038/s41379-019-0318-5 31285528

[66] Amary F Berisha F Ye H Gupta M Gutteridge A Baumhoer D H3F3A (histone 3.3) G34W immunohistochemistry: a reliable marker defining benign and malignant giant cell tumor of bone. Am J Surg Pathol (2017) 41(8):1059–68. 10.1097/PAS.0000000000000859 28505000 PMC5510691

[67] Antal I Sápi Z Szendröi M. The prognostic significance of DNA cytophotometry and proliferation index (Ki-67) in giant cell tumors of bone. Int Orthop (1999) 23(6):315–9. 10.1007/s002640050381 10741513 PMC3619848

[68] Rousseau MA Handra-Luca A Lazennec JY Catonné Y Saillant G. Metachronous multicentric giant-cell tumor of the bone in the lower limb. Case report and Ki-67 immunohistochemistry study. Virchows Arch (2004) 445(1):79–82. 10.1007/s00428-004-1011-7 15278449

[69] Alberghini M Kliskey K Krenacs T Picci P Kindblom L Forsyth R Morphological and immunophenotypic features of primary and metastatic giant cell tumour of bone. Virchows Arch (2010) 456:97–103. 10.1007/s00428-009-0863-2 20012988

[70] Ismail FW Shamsudin AM Wan Z Daud SM Samarendra MS. Ki-67 immuno-histochemistry index in stage III giant cell tumor of the bone. J Exp Clin Cancer Res CR (2010) 29(1):25. 10.1186/1756-9966-29-25 20226047 PMC2848627

[71] Parmeggiani A Micelli M Errani C Facchini G. State of the art and new concepts in giant cell tumour of bone: imaging features and tumour characteristics. Cancers (2021) 13(24):6298. 10.3390/cancers13246298 34944917 PMC8699510

[72] Chen S Du Z Wu B Shen H Liu C Qiu X STAT1, IGF1, RAC1, and MDM2 are associated with recurrence of giant cell tumor of bone. J Immunol Res (2018) 2018:4564328. 10.1155/2018/4564328 29651441 PMC5831922

[73] Metovic J Anaratone L Linari A Osella-Abate S Musuraca C Veneziano F Prognostic role of PD-L1 and immune-related gene expression profiles in giant cell tumours of bone. Cancer Immunol Immunother (2020) 69(9):1905–16. 10.1007/s00262-020-02594-9 32377818 PMC11027673

